# Immunological Evaluation of Recent MUC1 Glycopeptide Cancer Vaccines

**DOI:** 10.3390/vaccines4030025

**Published:** 2016-07-26

**Authors:** Md Kamal Hossain, Katherine A. Wall

**Affiliations:** Department of Medicinal and Biological Chemistry, University of Toledo, Toledo, OH 43606, USA; MdKamal.Hossain@rockets.utoledo.edu

**Keywords:** MUC1, adjuvant, BSA, KLH, TLR, liposome, vaccine

## Abstract

Aberrantly glycosylated mucin 1 (MUC1) is a recognized tumor-specific antigen on epithelial cell tumors. A wide variety of MUC1 glycopeptide anti-cancer vaccines have been formulated by many research groups. Some researchers have used MUC1 alone as an immunogen whereas other groups used different antigenic carrier proteins such as bovine serum albumin or keyhole limpet hemocyanin for conjugation with MUC1 glycopeptide. A variety of adjuvants have been used with MUC1 glycopeptides to improve their immunogenicity. Fully synthetic multicomponent vaccines have been synthesized by incorporating different T helper cell epitopes and Toll-like receptor agonists. Some vaccine formulations utilized liposomes or nanoparticles as vaccine delivery systems. In this review, we discuss the immunological evaluation of different conjugate or synthetic MUC1 glycopeptide vaccines in different tumor or mouse models that have been published since 2012.

## 1. Introduction

**Figure 1:**
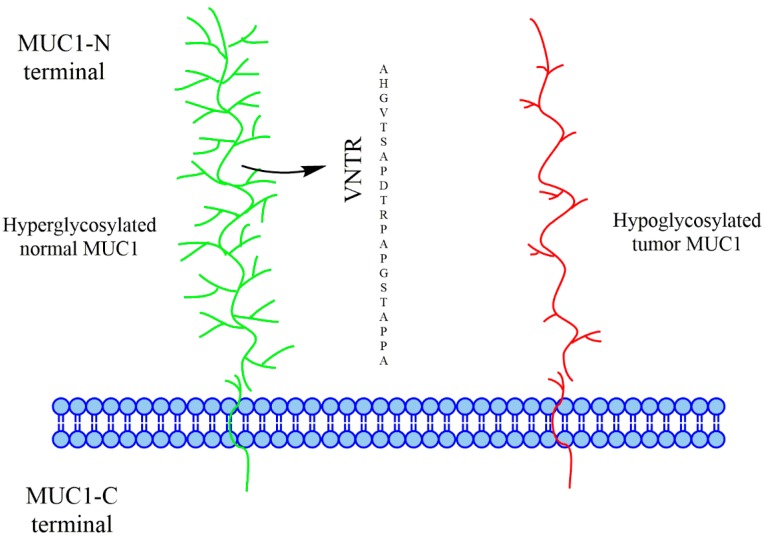
Difference between normal mucin 1 (MUC1) and tumor-associated MUC1.

The mucin 1 (MUC1) cell surface associated in humans is a transmembrane protein expressed in the lung, breast, pancreas, kidney, ovary, colon, and other tissues. It consists of the extracellular N-terminal domain containing a variable number of 20 amino acid tandem repeat (VNTR) units and the transmembrane and intracellular C-terminal region. In the core peptide portion of MUC1, each tandem repeat region contains five potential O-linked glycosylation sites on serine or threonine residues of MUC1 VNTR. It is highly glycosylated in normal cells, whereas in cancer cells, it is either hypoglycosylated or aberrantly glycosylated (Figure 1). This structural difference in MUC1 between normal and cancerous tissues makes it an attractive target for cancer immunotherapy. That is why the National Cancer Institute has placed MUC1 as a second prioritized cancer antigen for translational research [1].

Different types of carbohydrates contribute to form different antigens in aberrantly glycosylated or hypoglycosylated MUC1. The most common tumor associated carbohydrate antigens (TACAs) are Tn, Thomsen-Friedenreich (TF), sialyl Tn and sialyl TF [2]. Conjugation of *N*-acetylgalactosamine with a serine or threonine residue of MUC1 forms Tn, whereas addition of galactose forms TF. Tumors are deficient in core 1,3-galactosyl-transferase (T synthase) which causes the MUC1 to be aberrantly glycosylated and produce carbohydrate structures such as Tn (GalNAcα-Ser or -Thr), STn (Neu5Ac-α (2,6)-GalNAcα-Thr) and TF antigen (Gal-β (1,3)-GalNAcα-Thr) [3]. Aberrantly glycosylated MUC1 has shortened core-1 based glycans resulting from termination by sialyl groups that prevent cancer cells from forming core-2 based glycans, necessary to become hyperglycosylated MUC1. This happens either due to mutation of Cosmc chaperone of T synthase or down regulation of glycosyl transferase and up regulation of sialyl transferase. This makes the core peptide chain unable to produce core 2 or core 3 glycans and causes it to become antigenic [3].

In different cancers, the expression of glycosyltransferase enzymes in the ER and Golgi apparatus can vary and result in different glycolipid or glycoprotein structures. These enzymes can act as biomarkers for different types of cancers. For example, polypeptide *N*-acetylgalactosaminyltransferase (ppGalNAc-T) has been found to be a biomarker and prognostic indicator for breast, gastric and ovarian cancers [4,5,6,7]. *N*-acetylglucosamine transferases (GlcNAcT) have been proposed to have a role in invasion or metastasis in gastric and breast cancer as well as serving as biomarkers [8,9,10]. Multiple sialyltransferases have been associated with breast and colorectal cancer, with enhanced tumorigenicity, and with effects on prognostic indicators [11,12].

Immunologic tolerance is a very important issue for effective cancer vaccine preparation. Unlike bacterial cells, tumor cells have components such as glycolipids or glycoproteins which may be considered as self-antigens. The immune system may generate central and peripheral tolerance against them even after initial production of high numbers of CD8+ T cells [13,14]. Human MUC1 differs substantially from murine MUC1. Previous studies have found that mice transgenic for human MUC1 (MUC1 transgenic mice) are more prone to show tolerance against human MUC1 in comparison to wild type mice [15,16]. This complicates the development and testing of anti-cancer vaccines for the human antigen in mouse models.

Earlier attempts at immunization with nonglycosylated MUC1 were not successful as mice failed to produce enough anti-tumor cytotoxic T lymphocytes (CTL) and IgG due to lack of similarities between nonglycosylated and tumor-associated, aberrantly glycosylated MUC1 [17,18,19]. Also, heavily glycosylated MUC1 was not effective as a vaccine candidate due to its impaired susceptibility during antigen processing [20]. However, very recently it has been found that even nonglycosylated MUC1 peptide vaccines can produce CD8+ T cells in MUC1 transgenic mice that can recognize glycosylated MUC1 antigen [21].

## 2. Different Targeting Mechanisms and Strategies

In order to generate an effective anti-cancer vaccine response, the vaccine candidate should produce both humoral and cellular immunity [22]. Different types of MUC1-peptide vaccines have been synthesized chemically to produce effective anti-MUC1 immune responses. It has been found that using MUC1 alone does not produce strong immune responses, which necessitates the use of adjuvant and/or different T and B cell epitopes. For example, to prepare an efficient vaccine, MUC1 glycopeptide antigen has been conjugated either to additional T helper cell peptide epitopes or Toll like receptor (TLR) ligands to form two component vaccines or a combination of both to prepare three component vaccines [23].

CD4+ T helper cell epitopes are necessary to activate T helper cells that are required for antibody isotype switching as well as affinity maturation. They are also necessary for efficient memory B cell production required for future action [24]. Although having a variety of epitopes appears to be beneficial, a vaccine must not direct the immune response away from the tumor antigen. Adjuvants such as TLR ligands bind to TLR receptors and stimulate the release of inflammatory cytokines and chemokines necessary for stimulation of antigen presenting cells (APC) for activation of T helper cells. These cytokines and chemokines activate expression of co-stimulatory molecules for interactions among different T helper cells, B cells and APC [25]. There is a common tendency of cancer cells to evade immunosurveillance by induction of CD4+CD25+Foxp3+ regulatory T cells (Tregs), which may reduce the CTL response either through cell contact or by releasing suppressive cytokines [26,27,28,29]. It has been found that a TLR1/2 agonist can reduce the suppressive function of Tregs and enhance cytotoxicity of tumor-specific CTL in vitro and in vivo [30]. Thus, a TLR1/2 agonist would be an appropriate choice for effective anti-cancer immune therapy.

MUC1 targeted vaccines have employed MUC1 VNTR conjugated to different carriers such as Tetanus toxoid (TTox), keyhole limpet hemocyanin (KLH), and bovine serum albumin (BSA). [31,32,33,34,35,36,37,38]. Some vaccine preparations use different adjuvants such as bacillus Calmette-Guerin (BCG), monophosphoryl lipid A (MPL), Quillaja saponaria extract-21 (QS-21), or incomplete Freund’s adjuvant (IFA) to strengthen the immune response [32,33,39,40,41]. Other vaccines employed a recombinant adenovirus vector expressing MUC1 VNTRs to prime CD8+ cells followed by boosts of recombinant VNTRs in dimethyldioctadecylammonium liposomes containing MPL (DDA/MPL) to stimulate humoral responses [42]. MUC1-VNTR inserted into cholera toxin B subunit combined with aluminum hydroxide and CpG adjuvant generated a good immune response including CD8+ activity [43]. The MUC1 peptide conformation in this construct was designed to resemble the natural MUC1 conformation, ensuring a better MUC1 specific immune response in mice. Other techniques that have been exercised by researchers for MUC1 vaccines include liposome based cancer vaccines, targeting CD8+ T cells to stimulate CTL activity, dendritic cell vaccines loaded with peptide, and dendritic cell vaccines loaded with mRNA or transfected with cDNA [44]. In this review, we focus on the immunological aspects of the variety of new MUC1 conjugate or synthetic vaccines that have been indexed in PubMed from 2012 to the present.

## 3. MUC1 Glycopeptide with Different Protein Carriers

It has been popular for a long time to use protein conjugates as vaccine candidates prior to the use of fully synthetic multivalent cancer vaccines as immunotherapy. Different protein carriers such as BSA and KLH have been conjugated with MUC1 glycopeptide to produce immune responses, since these protein carriers are highly immunogenic and contain many epitopes. Cai et al. synthesized MUC1 with T or Tn antigen conjugated to BSA and found enhancement in the anti-MUC1 immune response [45]. The same group then developed MUC1 with STn or 2,6-STn conjugated to BSA and immunized BALB/c mice. Surprisingly, they found a strong IgG response capable of binding to MCF-7 tumor cells, whereas they and others found low immunogenicity in their earlier investigations with the BSA vaccine containing MUC1-STn [36,46].

Cai et al. then synthesized MUC1 glycopeptide conjugated either to BSA or to three different T-helper cell epitopes from TTox and immunized mice with the conjugate in buffer solution or Freund’s adjuvant [47]. Surprisingly they often found stronger immune responses to vaccines in buffer solution compared to vaccines in Freund’s adjuvant, particularly with regard to tumor binding antibodies and activation of complement dependent cytotoxicity (CDC). Freund’s adjuvant is no longer considered safe for human use because of severe side effects [48]. Hoffmann-Roder et al. synthesized a MUC1 glycopeptide-BSA conjugate vaccine containing 3’-fluoro-TF antigen, which generated anti-MUC1 mouse antibodies with specific binding to TF antigen [37]. They also reported synthesis of a 4’-fluoro-TF-MUC1-TTox/BSA conjugate vaccine, which produced IgG antibodies that bound to MUC1 epitopes on MCF-7 cancer cells [49].

Keyhole limpet hemocyanin (KLH) is another protein carrier that generates a strong immune response. Ragupathi et al. synthesized multivalent unimolecular vaccines conjugated to KLH that targeted breast and prostate cancer. Immunological data showed both IgG and IgM responses capable of binding with MCF-7 breast cancer cell lines expressing those multivalent antigens [50,51].

Tetanus toxoid (TTox) is another immunogenic carrier that has gained popularity due to its use in humans. Kaiser et al. synthesized MUC1-STn conjugated to TTox which induced a strong immune response in all mice [52]. Palitzsch et al. have recently reported synthesis of MUC1 glycopeptide vaccines with TTox as an immunogenic carrier [53]. Antibodies produced were mostly IgG1 with some IgM. They also generated a monoclonal antibody from an immunized mouse that showed strong binding to MUC1-expressing cancer cell lines T47D and MCF-7 and moderate binding to pancreatic cancer cell line PANC1, with no binding to non-cancerous human mammary epithelial cells.

However, most of the carrier proteins such as BSA and KLH seemed to be highly immunogenic by themselves and may have suppressed the antibody production against weak glycopeptide epitopes by feedback inhibition through Fc receptors on B cells [54,55,56,57]. That is one reason why fully synthetic multicomponent vaccine constructs having specific T helper cell epitopes combined with incorporated adjuvant have been analyzed in more recent studies.

## 4. Multicomponent Fully Synthetic MUC1 Vaccines

Two component fully synthetic MUC1 glycopeptide cancer vaccines have been synthesized by conjugating MUC1-VNTR including different TACAs either to various T helper epitope peptides, ovalbumin, or different TLR agonists. Cai et al. have reported synthesis of a multivalent vaccine containing tetravalent MUC1 VNTR sialyl-Tn antigen conjugated to TLR-2 ligand lipopeptide Pam3CysSK4. They have shown that more than 90 percent of MCF-7 cancer cells were killed when incubated with serum of mice given the tetravalent vaccine as compared to sera of those given other bi or tri-valent MUC1-sialyl-Tn antigen vaccines [58]. The isotype profile of the tetravalent vaccine reflected a higher IgG2a/IgG1 ratio and therefore a more Th1 type helper T cell response. [58].

Geraci et al. used calixarenes as a platform to build a multicomponent self-adjuvant vaccine by conjugating it with multiple units of the PDTRP MUC1 sequence [59]. Vaccines containing the PDTRP epitope produced more anti-MUC1 IgG antibodies compared to vaccines without PDTRP. These anti-MUC1 antibodies were able to recognize MUC1 expressing MCF-7 tumor cells. This vaccine did not contain known CD4+ T helper epitopes and presumably stimulated B cell responses by multivalent cross-linking of B cell receptors. The subclass distribution of IgG antibodies produced was not reported.

McDonald et al. reported use of macrophage activating lipopeptide 2 (MALP2) as an immuno-adjuvant and a TLR2/6 agonist in synthesizing a glycolipopeptide self-adjuvanting MUC1-MALP2 conjugate vaccine [60]. The activation of TLR2/6 receptors leads to downstream production of several cytokines and chemokines. MAPL2, as a stimulator of B cells, induces a specific antibody response without the help of an exogenous T helper cell epitope [60,61]. High titers of IgG1, IgG2b, IgG3 and IgM were generated without a measurable CD4+ or CD8+ T cell response.

Mono-phosphoryl lipid A (MPL) activates TLR4 and dimethyl dioctadecylammonium bromide (DDA) enhances antigen uptake and presentation. The combination of DDA/MPL has been reported to enhance antigen uptake, presentation to T cells and dendritic cell (DC) stimulation through TLRs [62,63,64,65]. Wang et al. used DDA/MPL as an adjuvant for preparing a recombinant MUC1 protein vaccine and found both cellular and humoral immune responses against MUC1 protein [42].

Yang et al. used C-type CpG oligodeoxynucleotide (YW002) along with MF59 as an adjuvant to boost the immunogenicity of recombinant fusion protein HSP65-MUC1 [66]. MF59 alone with HSP65-MUC1 could not inhibit the growth of MUC1+ B16 melanoma cells whereas MF59-YW002 did inhibit growth.

Three component fully synthetic MUC1 glycopeptide cancer vaccines have been synthesized by conjugating MUC1-VNTR with different TACAs serving as B cell epitopes along with various T helper cell epitopes and different TLR agonists as adjuvant. Lakshminarayanan et al. have reported that a three component vaccine containing aberrantly glycosylated MUC1 peptide, a T helper epitope peptide obtained from polio virus, and TLR-2 agonist Pam3CysSK4 elicited both humoral and cellular immunity [67]. They have also shown that a vaccine lacking only the Tn antigen (α-D-GalNAc) did not produce enough antibodies to induce antibody-dependent cellular cytotoxicity (ADCC) compared to the Tn-containing vaccine, proving that glycosylation is very important for antibody to recognize and initiate lysis of tumor cells. Also, this vaccine could effectively activate CD8+ T cells that recognized both glycosylated and nonglycosylated structures, whereas CD8+ T cells obtained after immunizing with the nonglycosylated vaccine only recognized the nonglycosylated structure. The CD8+ immune response was demonstrated by interferon-γ production and CTL killing of peptide-pulsed target cells, indicating cross presentation on MHC class I for CTL priming.

Abdel-Aal et al. have discussed the differences between the anti-MUC1 response resulting from the use of two different TLR agonists [68]. A TLR2 ligand (Pam3CysSK4) containing tripartite glycopeptide liposomal vaccine reduced the tumor burden more significantly than a TLR 9 ligand CpG oligodeoxynucleotide (CpG-ODN)-containing vaccine. Also, the Pam3CysSK4 containing vaccine reduced the tumor burden and generated both humoral and cellular immunity, neither of which was observed with the CpG-ODN-containing vaccine. TLR1/2 agonists were thought to stimulate the production of cytokines that inhibited the function of regulatory T cells commonly found in tumors.

Thompson et al. used sialyl-Tn antigen in a liposomal MUC1 glycopeptide vaccine along with the T helper cell epitope obtained from polio virus and Pam3CysSK4 [69]. It generated both humoral and cellular immunity against STn MUC-1 and tumor associated MUC1. MUC1 itself was shown to contribute to the T helper cell epitopes involved.

Very recently Cai et al. utilized gold nanoparticles (AuNP) as a carrier for a three component vaccine containing MUC1 glycopeptide with CD4+ T cell peptide epitopes (P-30) obtained from TTox [70]. This synthetic three component vaccine showed promising antibody titers, mostly IgG1, IgG2a and IgG2b, compared to a two component vaccine formulation (MUC1-P30) without AuNP, which generated mostly IgG1 and some IgM, even though the latter vaccine had ten times higher antigen concentration. This was interpreted to indicate that presentation of antigen through MHC was more effective in the case of the AuNP containing vaccine and this enhanced antibody production. Also, binding of antibodies with the breast cancer cell line MCF-7 showed that the antibodies could recognize cancer cells.

**Figure 2:**
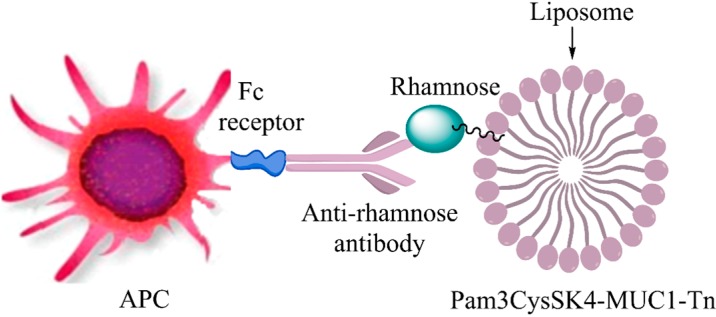
Antibody-mediated antigen uptake mechanism. Anti-rhamnose antibody binding to rhamnose on the vaccine allows recognition by Fc receptor (FcR) on antigen presenting cells and enhanced uptake.

Sarkar et al. have previously shown that vaccine immunogenicity can be augmented by coupling the bacterial monosaccharide rhamnose (Rha) to the vaccine. Naturally occurring anti-rhamnose antibodies in humans, or induced anti-rhamnose antibodies in mice, could bind the rhamnose and enhance vaccine uptake by APC (Figure 2). This was done initially with Rha-YAF-Tn, where YAF is a known CD4 helper T cell epitope coupled to Tn-Thr [71]. The Rha containing vaccine was more effective in mice bearing anti-Rha antibodies than in control mice. This same targeting concept was applied to MUC1 glycopeptide (Pam3Cys-MUC1-Tn) incorporated into L-rhamnose displaying liposomes to increase vaccine uptake and presentation on MHC. Again, antigen presentation was enhanced for both anti-tumor antibody production and generation of MUC1-reactive CD4+ T cells [72]. Karmakar et al. synthesized liposomes bearing a Pam3Cys-modified MUC1 glycopeptide containing B, CD4+, and CD8+ epitopes that was formulated with Rha-tetra(ethylene glycol)-cholesterol [73]. Fc-FcγR and other interactions enabled vaccine uptake and presentation of CD4+ epitopes on MHC class II and cross-presentation of CD8+ epitopes on MHC class I of the APC. Enhancement of both antibody production and CD8+-mediated cytotoxicity and interferon-γ production was observed. This indicated that antibody-assisted uptake could also increase cross-presentation of external antigens. An advantage of using endogenous MUC1 CD4+ and CD8+ epitopes was that the T cells generated could be re-stimulated at the site of the tumor by endogenous MUC1 peptides for cytokine production and CTL activity.

Hartmann et al. used nanohydrogel particles loaded with self-adjuvant CpG along with MUC-1 Tn and a TTox helper epitope peptide [74]. The CpG loaded nanogels upregulated the expression of co-stimulatory molecules CD40, CD80, and CD86 on APC, which is necessary for T cell proliferation. The CpG-loaded nanogels produced a higher antibody titer than nanogels without CpG. The antibodies also showed stronger binding to human breast cancer cell T47D.

Nuhn et al. examined antitumor vaccines containing the water soluble multi-valent carrier polymer poly(*N*-(2-hydroxypropyl)methyl acrylamide) P(HPMA) that allowed additional structures to bind and help in aggregation, ensuring better interaction with the immune system [75]. Their synthetic vaccine contained MUC1 glycopeptide and T helper cell epitope P2 conjugated with P(HPMA) polymer. Antisera from immunized mice showed IgG antibodies, mostly IgG1 and IgG2a, which indicated MHC-restricted T cell help. The antibodies showed notable binding to tumor cell MCF-7. Instead of using HPMA, Glaffig et al. used hyper-branched polyglycerol (HPG), an inert polymer which is water soluble and non-linear unlike HPMA and has a dendrimer-like structure that allows it to bind with multiple antigens and prevent entanglement of bound antigen [76]. In this case, a higher valency and more glycosylation gave better anti-tumor antibody responses.

Lu et al. introduced 12-mer MUC1-VNTR in place of the B cell epitope of cholera toxin B (CTB) to create CTB-presented MUC1 antigen (CTB-MUC1), thus attempting to keep the MUC1 peptide conformation in its native form [43]. Since CTB traps mucosal lymphocytes and macrophages and lowers the required antigen concentration for inducing an immune response, it is considered as a good carrier for inducing a mucosal immune response. Immunizing mice with CTB-MUC1 in aluminum hydroxide and CpG adjuvant (CTB-MUC1-Alum-CpG) produced a good CTL response. Also, high titers of IgG2c with a higher ratio of IgG2c/IgG1 indicated Th1 polarization.

Huang et al. synthesized several self-adjuvant vaccine candidates containing full length MUC1-VNTR and a self-assembly peptide sequence Q11 domain [77]. The Q11 domain acted as both adjuvant and carrier and aggregated into fibers. Those vaccine candidates contained multi-valent B cell epitopes on their surfaces and were assumed to elicit a stronger B cell response compared to a single B cell epitope in a vaccine. The vaccine elicited antibodies of IgG2a and IgM isotypes which could bind to MCF-7 cells and initiate CDC [78].

The synthesis of a four component vaccine containing three different T helper cell epitopes has been designed by Palitzsch et al. to ensure sufficient activation of T helper cells [24]. Antibody titers indicated that even though the vaccine did not have an external immune-stimulating adjuvant, it produced eight times more MUC1-specific antibodies, mostly IgG1 and some IgM, than two component vaccines containing only one T helper cell epitope. Antisera generated with the four component vaccine showed stronger binding to MUC1-expressing breast cancer cell line T47D as compared to that of antisera induced with two component vaccines.

Another unique approach to synthesizing an anti-tumor vaccine used multilayer self-assembly of components, with the T helper cell epitope as the core of the assembly, the immunostimulant γ-polyglutamic acid (γ-PGA) as the inner layer, and MUC1 glycopeptide as the outer layer, based on interactions between positive and negative charges [79]. The γ-PGA was included to stimulate APCs to produce cytokines. It elicited high levels of antibodies of predominantly IgG2a and IgM isotypes that bound to MCF-7 cells to initiate killing through CDC.

A DNA vaccine is another way to induce an immune response and these have been used in a variety of disease models such as autoimmune disorders and carcinomas. A DNA vaccine induces both cellular and humoral immune responses since it uses both extracellular and intracellular pathways for processing antigens [80]. However, DNA vaccines generally are weakly immunogenic if used without any booster or modification. Weng et al. designed a MUC1-DNA vaccine containing a 2-fold VNTR sequence as the target antigen to construct a plasmid. They showed that a pcDNA3.1-VNTR vaccine could induce both humoral and cellular immune responses in BALB/c mice and control growth of 653-MUC1 tumors [81].

Immunological responses to the various vaccine preparations are summarized in Table 1.

## 5. Use of Tn, STn and TF Analogs to Enhance Vaccine Stability

One of the main reasons for Tn on vaccines becoming less efficient is its sensitivity to the endogenous glycosidases that break down the TACA through enzymatic degradation and make it less bioavailable in vivo. Hence the structural modification of different TACAs has been seen as an approach to improve the immunogenicity of tumor associated antigens. Instead of using native tumor associated carbohydrate antigen, different groups have reported use of different mimetics or analogs of those antigens. Richichi et al. reported use of four clustered Tn antigen mimetics in place of native Tn antigen to improve its stability towards glycosidases. The mimetic elicited strong IgM and IgG responses, predominantly IgG2a and IgG1, that could recognize native Tn antigens [82]. Hoffmann-Roder et al. made TF antigen mimetics by replacing OH groups with two fluorine substituents in the 6 and 6′ positions of the pyranose rings. Both TF and TF mimetics generated comparably strong immune reactions [38,83]. Yang et al. synthesized 40 STn analogs and showed that some of the fluorine-containing STn analogs elicited better anti-STn IgG titers and could detect native STn antigen-expressing tumor cells [84,85]

## 6. Conclusions

As described above, a wide variety of approaches have been used to generate immune responses to MUC1. Most of these approaches are sufficient to stimulate the production of antibodies that can bind to TACAs on human tumor cells. In many cases, these antibodies have been shown to activate CDC and ADCC.

Earlier studies focused on providing exogenous helper T cell epitopes to facilitate isotype switching. More recently, endogenous MUC1 CD4+ epitopes have been used to provide T cell help. Only a few studies have directly demonstrated the generation of MUC1-specific CD4+ cells. Efficient tumor elimination also utilizes TACA-specific CD8+ cytotoxic T cells, particularly since tumor-shed, aberrantly glycosylated MUC1 can interfere with tumor targeting by anti-MUC1 antibodies. In most cases, priming of MUC1-specific CD8+ T cells is thought to be through cross-presentation of vaccine glycopeptide CD8+ epitopes on MHC class I. The tumor-specific MUC1 peptides presented on class I MHCs of tumor cells are hypoglycosylated. This complicates the priming of a CD8+ response by live vaccines or APC transfected with MUC1, which would produce MUC1 peptides with normal glycosylation. This may be an impediment to developing DNA or viral MUC1 vaccines intended to produce cellular anti-tumor responses. For these reasons, synthetic glycoconjugate vaccines are an appropriate avenue for anti-MUC1 cancer vaccine development.

## Figures and Tables

**Table 1:** Different strategies for producing anti-MUC1 immunological responses.

Authors	Year	Vaccine Preparations	Cell Lines & Animals	Response
Karmakar et al. [73]	2016	Rhamnose containg liposomal Pam3Cys-MUC1-Tn	EL4, C57BL/6	CTL, IFN-γ
Lakshminarayanan et al. [21]	2016	Glycosylates, no-glycosylates or tumor derived MUC1 vaccines	C57mg, MC38, B16, EL4, Panc02, MUC1 Tg mice (C57BL/6)	IFN-γ producing CD4+ & CD8+ cells
Cai et al. [70]	2016	Glycopeptide-functionalized gold nanoparticles	MCF7, BALB/c	IgG1, IgG2a, IgG2b
Lu et al. [43]	2015	MUC1 peptide on cholera toxin B (CTB) subunit	B16, C57BL/6	CTL, Th1
Glaffig et al. [76]	2015	MUC1-P2 conjugate on hyperbranched polymers	T47D, BALB/c	IgG1, IgG2a, IgG2b, IgM
Thompson et al. [69]	2015	MUC1-STn glycopeptide with T cell epitope & TLR2 ligand	C57mg, B16, MUC1 Tg mice (C57BL/6)	CTL, IFN-γ, IgG1, IgG2a, IgG2b, IgG3
Hartmann et al. [74]	2015	CpG loaded nanohydrogel particles	T47D, BALB/c	Upregulation of CD40, CD80, CD86
Johannes et al. [49]	2015	Fluorinated MUC1-BSA/TTox conjugate vaccine	MCF7, BALB/cj	IgG1, IgG2a, IgG2b but no IgM
Wang et al. [42]	2014	MUC1 VNTR and (MUC1-VPP) with DDA/MPL as adjuvant	B16, C2C12, C57BL/6	CTL, IFN-γ
McDonald et al. [60]	2014	MUC1-MALP2 conjugate vaccine	C57BL/6	IgM, IgG1, IgG2b, IgG3
Cai et al. [58]	2014	Multivalent glycopeptide-lipopeptide vaccine	MCF7, BALB/c	IgG, IgM
Palitzsch et al. [24]	2014	MUC1 glycopeptide with three different Th cell epitopes	T47D, BALB/c	IgG, IgM
Abdel-Aal et al. [68]	2014	MUC1 tripartite vaccine modified by TLR 2 or TLR 4	C57mg, EL4, MUC1 Tg mice (C57BL/6)	IgG, IgM, CTL
Geraci et al. [59]	2013	PDTRP MUC1 conjugated calixarene containing TLR2 ligand	MCF7, BALB/c	IgG
Sarkar et al. [72]	2013	MUC1 glycopeptide into L-Rhamnose displaying liposomes	BALB/c	IgG1, IgG2a, IgG2b, IgM
Cai et al. [47]	2013	MUC1 glycopeptide with T cell epitope from TTox	MCF7, T47D,	IgG1, IgG2a, IgG2b, IgG3, IgM
Nuhn et al. [75]	2013	MUC1-VNTR with T helper epitope and P(HPMA)	MCF7, BALB/c	IgG
Yang et al. [66]	2012	Recombinant HSP65-MUC1 and MF59-YW002 as adjuvant	B16, C57BL/6	Th1, CTL
Huang et al. [77]	2012	MUC1 glycopeptide with a B cell epitope	MCF7	IgG1, IgG2a, IgG2b, IgG3, IgM
Cai et al. [36]	2012	MUC1 Glycopeptide-BSA vaccine	MCF7, BALB/c	IgG1, IgG2a, IgG2b, IgG3, IgA, IgM
Weng et al. [81]	2012	MUC1-2-VNTR DNA vaccine	BALB/c	CTL
Lakshminarayanan et al. [67]	2012	MUC1 tripartite vaccine	Yac.MUC1, C57mg, MUC1 Tg mice (C57BL/6)	IgG, CTL, TNF-α, RANTES, IL-6, 12, IL-1β

## Abbreviations

The following abbreviations are used in this manuscript:

ADCC	Antibody-dependent cellular cytotoxicity
APC	Antigen presenting cells
AuNP	Gold nanoparticles
BCG	Bacillus Calmette-Guérin
BSA	Bovine serum albumin
CDC	Complement-dependent cytotoxicity
CTB	Cholera toxin B subunit
CTL	Cytotoxic T lymphocyte
DDA	Dioctadecylammonium bromide
IFA	Incomplete Freund’s adjuvant
KLH	Keyhole limpet hemocyanin
MHC	Major histocompatibility complex antigen
MPL	Monophosphoryl lipid A
MUC1	Mucin 1
ODN	Oligodeoxynucleotide
P(HPMA)	Poly(*N*-(2-hydroxypropyl) methacrylamide
QS-21	Quillaja saponaria extract 21
Rha	Rhamnose
TACA	Tumor associated carbohydrate antigen
TF	Thomsen-Friedenreich
TLR	Toll-like receptor
Tregs	Regulatory T cells
TTox	Tetanus toxoid
VNTR	Variable number tandem repeat
